# The effects of continuous care model using a smartphone application on adherence to treatment and self-efficacy among patients with multiple sclerosis

**DOI:** 10.1186/s12911-022-01785-x

**Published:** 2022-02-26

**Authors:** Seyed Mojtaba Kazemi, Mahnaz Rakhshan, Mozhgan Rivaz, Sadegh Izadi

**Affiliations:** 1 Student Research Committee, School of Nursing and Midwifery, Shiraz University of Medical Sciences, Shiraz, Iran; 2 Community Based Psychiatric Care Research Center, School of Nursing and Midwifery, Shiraz University of Medical Sciences, Shiraz, Iran; 3 Department of Nursing, School of Nursing and Midwifery, Shiraz University of Medical Sciences, Shiraz, Iran; 4 Clinical Neurology Research Center, Shiraz University of Medical Sciences, Shiraz, Iran

**Keywords:** Multiple sclerosis, Adherence to treatment, Self-efficacy, Cell-phone, Application, Continuous care model, Telemedicine

## Abstract

**Background:**

Adherence to disease-modifying therapy is important in patients with Multiple Sclerosis (MS) to increase the positive outcomes and improve the quality of life. This study aimed to determine the effects of Continuous Care Model (CCM) using a smartphone application on adherence to treatment and self-efficacy among MS patients.

**Methods:**

This quasi-experimental study with pre/posttest design was conducted on 72 MS patients in Shiraz, Iran from June 2020 to August 2021. The samples were randomly assigned to intervention (n = 36) and control (n = 36) groups. In the intervention group, the CCM using a smartphone application was implemented during two months. However, no intervention was performed for the control group. The data were collected using the self-report Multiple Sclerosis Treatment Adherence Questionnaire (MS-TAQ) and MS Self-Efficacy Scale (MSSS) at baseline and two and four months after the intervention.

**Results:**

The results showed an improvement in adherence to treatment and self-efficacy in the intervention group compared to the control group after implementing the virtual CCM and at the two-month follow-up (*p* < 0.001).

**Conclusions:**

Implementing the CCM using a smartphone application resulted in improvements in the MS patients’ adherence to treatment and self-efficacy. It can be concluded that providing care using an interactive multimedia application can improve the outcomes as well as patients’ satisfaction, especially during the COVID-19 pandemic. Therefore, this approach is recommended to be used for nurses, healthcare providers, and clinicians.

**Supplementary Information:**

The online version contains supplementary material available at 10.1186/s12911-022-01785-x.

## Background

Multiple Sclerosis (MS) is the most prevalent chronic, inflammatory, destructive, and progressive demyelinating disease of the central nervous system [1]. It has been estimated that 2–5 million people suffer from MS around the world. In Iran, averagely 80,000 individuals suffer from MS [2], with the highest prevalence rates being related to Tehran, Isfahan, and Fars provinces with 102, 106.46, and 78 individuals per 100,000 population, respectively [3, 4]. MS significantly influences patients’ daily activities and quality of life [5, 6].

Dealing with chronic diseases usually requires long-term treatment plans to empower patients [7], which include Disease-Modifying Therapies (DMTs) (supportive care, rehabilitation, and symptom management) [8]. Adherence to DMTs is essential to maximize the beneficial effects of MS treatment and reduce the number of clinical relapses [9]. On the other hand, failure to follow treatment and care regimens increases the risk of complications and mortality as well as the cost of healthcare [10]. According to the World Health Organization (WHO), compliance with treatment regimens is an important factor in the success of treatment. Poor compliance reduces the desired clinical effects and, consequently, reduces the effectiveness of health systems [11]. Various factors are associated with adherence to treatment including age, gender, socioeconomic status, limited effectiveness of treatments, type of MS, patient’s attitude, side effects of drugs, amnesia, depression, anxiety, and cognitive problems. Identification and elimination of these factors increase adherence to treatment and help choose the effective treatment for MS [9]. Erbay et al. [12] reported an adherence rate of 59.6% in MS patients.

Decreased self-efficacy is another problem faced by these patients [13]. Self-efficacy is the main prerequisite for behavior change including health behaviors [14]. Sikes et al. [15] reported that MS could reduce self-efficacy by up to 48%. Considering the chronic nature of MS, self-efficacy is an important internal factor for long-term control and management of this disease [16].

Despite the recent therapeutic advances, there is still no known cure for MS [17]. Consequently, continuous care seems to be necessary to prevent the complications and recurrence of the disease [18]. The Continuous Care Model (CCM) aims to establish an effective, interactive, and consistent relationship between the client and the nurse as a provider of healthcare services to evaluate the clients’ needs and problems and sensitize them to accept their continuous health behaviors and help maintain their recovery process and health promotion, which is compatible with the characteristics of chronic diseases and the dynamics of their problems [19]. The recommended treatment regimen often takes place at home and outside the scope of medical care in patients with MS [20]. Thus, providing remote healthcare services increases the access of patients with mobility or geographical limitations [21]. Nowadays, access to health services can be greatly facilitated due to the advances in computer and network technologies [22].

Educational software programs create a participatory platform and provide valuable information, thereby creating an opportunity to improve the disease process that allows patients to access the required information upon request [23]. Therefore, it is important to educate clients through multimedia, as an appropriate method to address the educational needs of patients with MS [24]. The main advantages of this method include the multimedia feature or using a combination of texts and audios/videos, comprehensive activation, reproducibility, and feedback [25]. Evidence has also shown that multimedia software-based education can improve knowledge and compliance with treatment regimens [26].

As mentioned earlier, continuous care aims at designing and maintaining a flexible, dynamic and continuous care relationship between the nurse and the patient for improving patient outcomes. Thus, this model can lead to acceptance, increase of insight, and continuous engagement [27]. The introduction of this model using an Android-based smartphone application seems to be a good option for providing services to MS patients, particularly during the COVID-19 pandemic. Up to now, few studies have been published on the effect of this remote model on self-efficacy and adherence to treatment among MS patients. Therefore, the present study aimed to evaluate the effects of CCM using a smartphone application on adherence to treatment and self-efficacy among MS patients.

## Methods

### Study design

This quasi-experimental study with the pre/posttest design was conducted on 72 MS patients referred to the MS Association affiliated to Shiraz University of Medical Sciences, Shiraz, Iran from June 2020 to August 2021.

The study sample size was estimated according to a similar study [28] and considering α = 0.05, β = 0.2, and attrition rate of 15%. Patients were recruited via convenience sampling and were then randomly assigned to the intervention (n = 36) and control (n = 36) groups through block randomization with the block size of four using the Random Allocation Software. The inclusion criteria were suffering from relapsing–remitting MS, aging 18–45 years, having mild to moderate disability (EDSS 0-5-5), not being in the recurrence phase, being able to use smartphones, having an Android smartphone, and having the history of MS for at least six months. The exclusion criteria were suffering from other types of MS (primary progressive, secondary progressive and progressive relapsing), unwillingness to continue cooperation, incomplete attendance in educational interventions, severe disease complications, and known psychophysical disorders.

### Data collection

Data collection tools consisted of a demographic questionnaire, the MS-Treatment Adherence Questionnaire (MS-TAQ), and MS Self-Efficacy Scale (MSSS).

### Measurement

#### Demographic information form

The demographic information form included two parts, namely demographic characteristics (age, gender, marital status, education level, and occupation) and disease-related information (duration of the disease, frequency of recurrence, and number of hospitalizations during the past year).

#### MS-Treatment Adherence Questionnaire

The MS-TAQ is a self-report tool for identifying barriers to adherence amongst MS patients taking DMTs. The MS-TAQ was developed by Wicks et al. [29] in the United States. It contained 30 items divided into three subscales, namely DMT-Barriers, DMT-Side Effects, and DMT-Coping Strategies. In the DMT-Barriers subscale, the patients who had missed at least one dose in the previous 28 days were asked 13 four-point questions pertaining to the barriers to adherence (“not important at all” to “extremely important”). In the DMT-Side Effects subscale, 10 treatment side effects that might have a negative effect on adherence to treatment were listed in form of five-point questions from “never” to “all or nearly all the time.” The DMT-Coping Strategies included seven yes/no questions regarding the coping strategies that might be effective in reducing the treatment side effects. The reliability of this tool was confirmed by Cronbach’s alpha coefficients ranging from 0.40 to 0.86. In addition, the convergent validity of this questionnaire was reported as the relationship between Missed Dose Ratio (MDR) and the treatment adherence subscale (r = 0.5). Its divergent validity was also reported as the relationship between coping strategies and MDR (r = 0.3). The Persian version of the scale was validated by Maghsoud Puryousef [30] with a Cronbach’s alpha coefficient of 0.87. In the present study also, the Cronbach’s alpha of this instrument was computed as 0.90.

#### MS self-efficacy scale

MSSS was developed by Rigby et al. [31] in England. The MSSS included 14 items divided into four dimensions, namely independence and activity (five items), concerns and interests (four items), personal control (three items), and social confidence (two items). The items could be scored via a six-point Likert scale ranging from strongly disagree (1) to strongly agree (6). Thus, the total score could range from 6 to 84, with higher scores representing higher self-efficacy. The total score of the scale could be calculated through summing up the scores of the subscales. The reliability of the scale was approved by Cronbach’s alpha coefficient of 0.81. Moreover, the results of Exploratory Factor Analysis (EFA) indicated that the four-factor structure with 14 items explained 57.9% of the variance, which revealed the acceptable validity of the instrument. In Iran, the psychometric properties of MSSS were measured by Reshvanloo [32]. The validity of the scale was explored via construct validity (EFA) and divergent validity. Divergent validity was measured with a depression scale (r =  − 0.74). Additionally, the reliability of the whole scale was confirmed by Cronbach’s alpha = 0.9. In the present study, the Cronbach’s alpha was calculated as 0.92.

#### Development of the smartphone application

At first, educational materials on MS were extracted from authentic resources (i.e., textbooks, literature, etc.) and were evaluated and revised by two experts in the field of nursing and one neurologist. The educational content included the description of MS (i.e., pathophysiology, clinical manifestations, assessment and diagnosis, pharmacologic therapies, management of the disease, nutrition, pregnancy, exercise, etc.), COVID-19 and its prevention among MS patients, and self-management programs. Then, based on educational materials, electronic contents, images, animations, and audio and video clips were produced and designed in form of an installable application on electronic devices using an Android Studio program on the Android platform. Finally, the developed Persian multimedia software named the “MS App” was evaluated and tested by electronic content production engineers.

The “MS App” consisted of various sections including educational sections about the disease, injection training (i.e., interferon beta-1a and interferon beta-1a), entertainment section (i.e., motivational contents, music, etc.), night section (patients' night rest including audio podcasts, e-books, and instrumental music), chat room (for the patients to communicate with each other), and a notification system status bar. Besides, a section of the software was designed to interact with the researcher, so that the patients could communicate with the researcher whenever they needed. The software could be easily installed on Android smartphones and the contents were updated daily without the need to reinstall a new application (Additional file 1).

#### Intervention

Initially, the written informed consent form and the questionnaires were completed by both groups in the MS Association before the intervention. Then, the CCM using an Android-based interactive smartphone application was performed for the patients in the intervention group for 4 months. However, the control group only received the routine care. The CCM is a nursing care model that was developed and assessed psychometrically in patients with chronic coronary artery disease by Ahmadi et al. in Iran in 2001. This model consisted of four steps including orientation, sensitization, control, and evaluation [27].

The first stage (orientation) included explaining the aims, making a relationship with the clients, explaining the study protocol, collaboration to take part in the study, and involvement of the clients and their families in care. For this purpose, a 30–45-min session was held with the presence of the patients and their families at the clinic to identify the patients’ problems accurately motivate them to participate, explain the importance of continuous care, determine their expectations from each other, and express the need for continuation of the cooperation until the end of the study. At the end of the orientation session, the demographic information form, MS-TAQ, and MSSS were completed.

*Sensitization stage* Interventions were performed in this stage during two mouths. In doing so, sensitizing the clients to accept responsibility for their health was emphasized by evaluating their educational needs. Overall, the patients became familiar with the process of the disease, its complications, DMTs, adherence to treatment, and self-management programs and their possible questions were addressed through the MS App. This multimedia app could be easily installed on Android mobile phones.

*Control stage* This phase consisted of the assessment and continuity of care. Continuous care consultations and care needs were followed daily and weekly through a part of the MS App that was designed for this purpose. The telephone number of the researcher was given to the patients, as well.

*Evaluation stage* In the fourth stage, the effects of the interventions and follow-ups were evaluated using the scales for measuring and comparing adherence to treatment and self-efficacy.

Immediately after the intervention and at the two-month follow-up, the MSSS and MS-TAQ were completed by the two groups in an online platform due to the COVID-19 pandemic. In addition, the patients in the intervention group were requested to take part in an online survey and express their satisfaction with the implementation of the virtual CCM. At the end of the study, the MS App was made available to all the patients in the MS Association, Shiraz, Iran.

### Data analysis

The data were analyzed by the SPSS 22 software at the significance level of *p* < 0.05. Kolmogorov–Smirnov test showed the normal distribution of the data related to adherence to treatment and self-efficacy (*p* > 0.05). Thus, the data were analyzed using descriptive indices such as mean and Standard Deviation (SD) and inferential statistics including chi-square, Fisher’s exact test, independent *t*-test, repeated measures ANOVA, and LSD post-hoc test.

## Results

The results indicated that 66.7% of the patients were female and 48.6% were within the age range of 18–29 years. In addition, 62.5% of the patients were suffering from the disease for 1–5 years and 48.6% had a positive history of relapse. There were no significant differences between the intervention and control groups in terms of sociodemographic and clinical characteristics (*p* > 0.05) (Table 1).

**Table 1:** Sociodemographic and clinical characteristics of the samples

Group variable	Control (n = 36)		Intervention (n = 36)		Total (n = 72)		*P*-value*
	Number	Percent	Number	Percent	Number	Percent	
Gender^a^							
Male	15	41.7	9	25	24	33.3	0.8
Female	21	58.3	27	75	48	66.7	
Age^b^							
18–29	18	50	17	47.2	35	48.6	1
30–40	16	44.4	17	47.2	33	45.8	
40–45	2	5.6	2	5.6	4	5.6	
Educational level^a^							
Primary and intermediate school	12	33.3	11	30.6	23	31.9	0.87
High school	15	41.7	17	47.2	32	44.4	
University	9	25	8	22.2	17	23.7	
Marital status^a^							
Single	17	47.2	16	44.5	33	45.8	1
Married	19	52.8	20	55.6	39	54.2	
Job^a^							
Unemployed	9	25	11	30.6	20	27.7	0.8
Employed	10	27.8	6	16.7	16	22.3	
Housewife	17	47.2	19	52.8	36	50	
Disease duration^b^							
Under one year	2	5.6	1	2.8	3	4.2	0.69
1–5 years	21	58.3	24	66.7	45	62.5	
5–10 years	7	19.4	8	22.2	15	20.8	
Above 10 years	6	16.7	3	8.3	9	12.5	
Relapse recurrence^a^							
Without relapsing	13	36.1	14	38.9	27	37.5	0.94
Once relapsing	17	47.2	18	50	35	48.6	
Twice relapsing	6	16.7	4	11.1	10	13.9	

^a^Chi-square test

^b^Fisher exact test

The results of between-group and within-group comparisons of the mean scores of adherence to treatment before, after, and two months after the intervention have been presented in Table 2. The results of independent t-test showed no significant difference between the two groups regarding the mean score of adherence to treatment before the intervention (*p* = 0.83). However, this difference was significant immediately and two months after the intervention. Repeated measures ANOVA was used to compare the mean scores of adherence to treatment in each group before, immediately after, and two months after the intervention. In the intervention group, a significant difference was observed in this regard before, immediately after, and two months after the intervention (*p* < 0.0001) (Table 2).

**Table 2:** Comparison of the mean scores of adherence to treatment before, after and two months after the intervention in between-group and within-group

Variable	Groups	Before intervention Mean ± SD	After intervention Mean ± SD	2 month after intervention Mean ± SD	*P*-value Within group
Adherence to treatment	Control	71.27 ± 5.64	71.33 ± 7.4	70 ± 3.97	0.539**
	Intervention	71.55 ± 5.68	20.08 ± 3.17	26.94 ± 2.97	*P* < 0.0001**
*P*-value between groups		0.836*	*P* < 0.0001*	*P* < 0.0001*	

^*^Independent *t*-test

^**^Repeated measures test

The results of between-group and within-group comparisons of the mean scores of self-efficacy before, immediately after, and two months after the intervention have been presented in Table 3. The results of independent *t*-test showed no significant difference between the two groups concerning the mean score of self-efficacy before the intervention (*p* = 0.80). However, this difference was significant immediately and two months after the intervention. Repeated measures ANOVA was used to compare the mean scores of self-efficacy in each group before, immediately after, and two months after the intervention. In the intervention group, a significant difference was observed in this regard before, immediately after, and two months after the intervention (*p* < 0.0001) (Table 3). LSD post-hoc test was used to assess the changes in different stages of the research. Furthermore, the findings indicated that the majority of the patients in the intervention group were satisfied with this approach.

**Table 3:** Comparison of the mean scores of self-efficacy before, after and two months after the intervention in between-group and within-group

Variable	Groups	Before intervention Mean ± SD	After intervention Mean ± SD	2 month after intervention Mean ± SD	*P*-value Within group
Self-efficacy	Control	30.41 ± 5.09	30.13** ± **3.49	27.83 ± 3.97	0.010**
	Intervention	30.69 ± 4.29	52.97** ± **4.01	44.91 ± 2.97	*P* < 0.0001**
*P*-value between groups		0.803*	*P* < 0.0001*	*P* < 0.0001*	

^*^Independent *t*-test

^**^Repeated measures test

## Discussion

The present study results indicated that applying the CCM using the MS App improved adherence to treatment and self-efficacy among the MS patients. Accordingly, the patients who received the MS App showed better levels of adherence to treatment compared to those in the control group immediately after the intervention and at the two-month follow-up. In fact, the mean score of adherence to treatment decreased by removing the barriers. Thus, obtaining a lower score indicated a better improvement in adherence to treatment. Previous studies demonstrated that the CCM was effective in treatment adherence among hemodialysis and myocardial infarction patients [33, 34] and affected lifestyle modification in patients with MS [35]. Similarly, Golan et al. [36] reported that an electronic notebook through smartphones could improve treatment adherence amongst MS patients.

In the present study, after applying the continuous care model and at the two-month follow-up, the mean score of self-efficacy was higher in the intervention group than in the control group. A recent study also concluded that the continuous care model of information-based hospital-family integration via online social media could improve self-efficacy, colostomy complications, and quality of life in colostomy patients [37]. In the same vein, Ehde et al. indicated the effectiveness of telephone-based self-management interventions in improving self-efficacy and reducing the complications of MS patients. They came to the conclusion that the telephone-delivered intervention was effective in engaging patients in care and improving their disabilities [38]. Another study also revealed that having access to smartphone apps enhanced women’s performance, self-efficacy, and health beliefs in breast self-examination [39]. Evidence has shown that during the COVID-19 outbreak, smartphones and apps have played a key role in several aspects of healthcare delivery and clinical practice among healthcare professionals [40, 41]. Therefore, alternative approaches can facilitate healthcare delivery during the pandemic.

### Strengths and limitations

A key strength of the current study was the innovation in applying continuous care for the MS patients during the COVID-19 pandemic. Implementing the remote CCM during the pandemic minimized the patients’ face-to-face visits and consequently, reduced the risk of spread of the viral infection among patients, healthcare providers, and clinicians, and increased patient satisfaction. However, the study had several limitations including the selection of patients with a specific type of MS (relapsing–remitting), which might increase the likelihood of bias in the data. Another study limitation was selecting the samples within the age range of 18–45 years. With smartphone applications, technology is more likely to be embraced by the younger generation, thus limiting its applicability in older individuals. As another limitation, adherence to treatment was measured using self-report scale that did not directly assess adherence and had a weak correlation (r = 0.5) with the missed dose ratio. Other study limitations included the time-consuming design of the application, the outbreak of COVID-19 that prolonged the sampling process, and the short follow-up period (two months) due to the limited research time.

## Conclusions

The results demonstrated that implementing the CCM using the MS App led to improvements in treatment adherence and self-efficacy among the MS patients. Thus, it can be concluded that providing care with an interactive multimedia application can enhance the outcomes as well as patient satisfaction, especially during the COVID-19 pandemic. Therefore, this approach is recommended to be used for nurses, healthcare providers, and clinicians. This approach can also be used to provide continuous care in other interventions for MS patients. However, its usefulness is required to be assessed in further studies.

## Supplementary Information

**Additional file 1**. The Persian “MS App” consisted of various sections including main menu, MS introduction, beyond MS, entertainment section, and night episode.

## Data Availability

The datasets used and analyzed during the current study are available from the corresponding author on reasonable request.

## Abbreviations

CCM: Continuous care model
COVID-19: Coronavirus disease of 2019
MS: Multiple sclerosis
RR: Relapsing–remitting
MS-TAQ: Multiple sclerosis treatment adherence questionnaire
MSSS: Multiple sclerosis self-efficacy scale
MD: Mean difference
DMT: Disease-modifying therapy
