# Supplementary material for: The effects of continuous care model using a smartphone application on adherence to treatment and self-efficacy among patients with multiple sclerosis
Source: BMC Med Inform Decis Mak. 2022 Feb 26;22:53. doi: 10.1186/s12911-022-01785-x (PMC8881942; doi:10.1186/s12911-022-01785-x)
Supplement: Supplementary file 1 — Additional file 1. The Persian “MS App” consisted of various sections including main menu, MS introduction, beyond MS, entertainment section, and night episode. [file 12911_2022_1785_MOESM1_ESM.pdf]

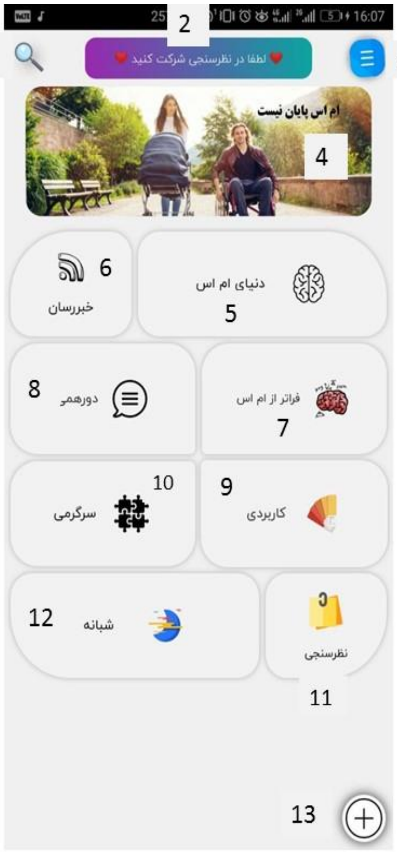

Main Menu

1-Slide Menu
2-During the intervention, this section contained important points about MS
3-Search Button
4-Image Slider(Contains motivational images)
5-Button to enter the MS introduction section
6-NEWS section (displays daily news for patients, including about MS in different categories)
7-Additional information about MS such as daily injections of drugs and …
8-Chatroom
9-The Efficiency part includes online visits and …
10-The entertainment section includes games, motivational content and …
11-In this section, there was a link to the questionnaires
12-In this section, there is relaxing content for patients to sleep
13- Quick menu button for easy access to different parts of the program

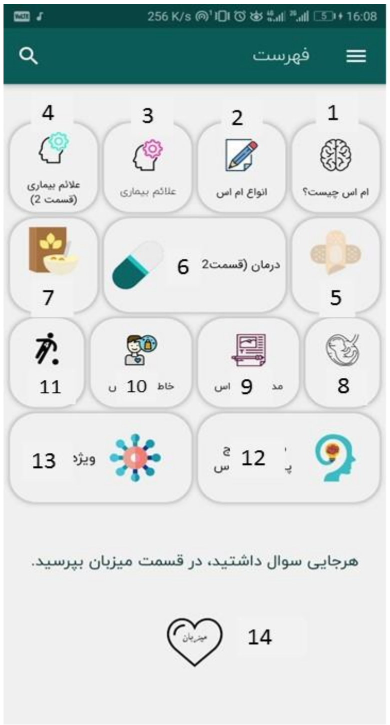

MS Introduction

1-MS introduction
2-Types of MS
3-introduction of Signs of MS
4-Extra signs of MS
5-Types of treatments
6-Extra ways of treatments
7-nutritions
8-pregnancy during MS
9-Tips for self management
10-Recommendations from other people with MS
11-activities in MS
12-Frequently Asked Questions About MS
13- Sub-Special Tips about COVID-19 and MS
14-Communication with the researcher

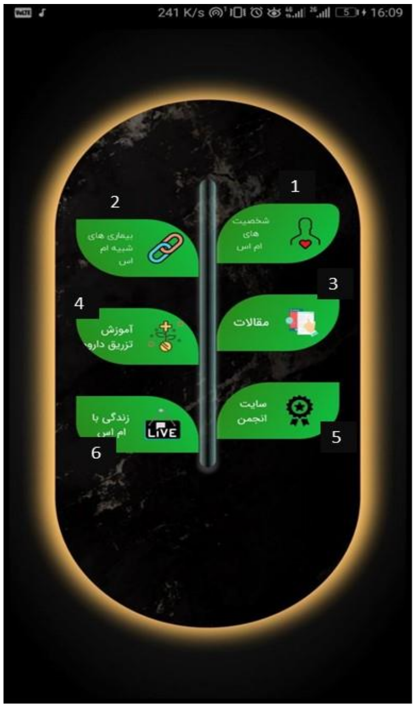

beyond MS

1-Introducing successful characters and people with MS
2-Introduction of diseases similar to MS
3-Introducing the results of updated articles about MS
4- MS drug injection training
5-Introducing the MS Association Website
6-MS lifestyle

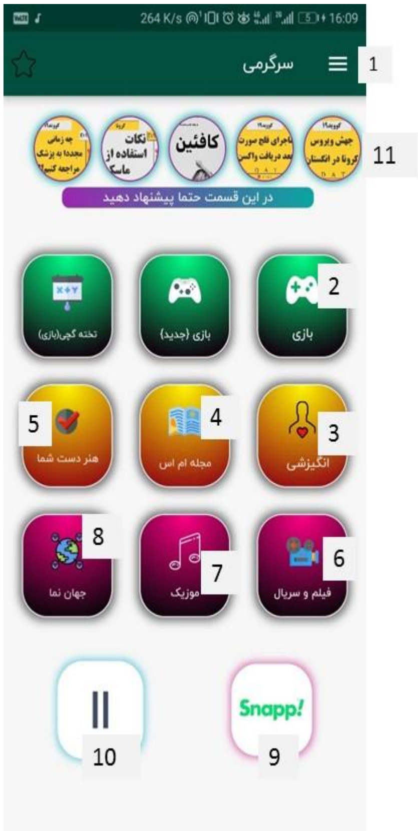

Entertainment section

1-Slide Menu
2-Game
3-Motivational content includes text, e-books, movies and podcasts
4- An online magazine published by the Iranian MS Association
5-Introducing different skills of MS patients
6-Movie and Serials
7-Music
8-Live view of Shiraz tourist areas
9-Request an online taxi
10-Online Radio Music
11-This section contains various photos about what you know today, which is similar to the Instagram story section. This section was updated daily by the researcher with new content.

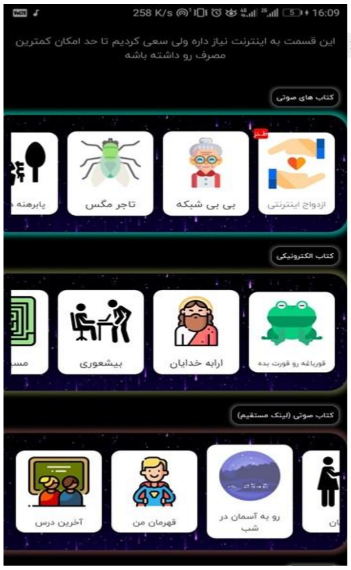

Night episode

This section includes audio books, e-books, non-verbal music (for relaxation before bed).

Caption:

Additional file 1: App Images: The Persian "MS App" consisted of various sections including main menu, MS introduction, beyond MS, entertainment section, and night episode.
